# Retroperitoneal Inflammatory Liposarcoma in a Patient with Non-Hodgkin Lymphoma: A Report Highlighting Diagnostic Pitfalls

**DOI:** 10.4061/2010/505436

**Published:** 2010-12-05

**Authors:** Cathy S. Lim, Caroline L. Cooper, Warick Delprado, James Kench, Stanley W. McCarthy, Richard A. Scolyer

**Affiliations:** ^1^Tissue Pathology and Diagnostic Oncology, Royal Prince Alfred Hospital, Camperdown, NSW 2050, Australia; ^2^Department of Histopathology, Douglass Hanly Moir Pathology, 14 Giffnock Avenue Macquarie Park, Sydney, NSW 2113, Australia; ^3^Melanoma Institute Australia, 40 Rocklands Road, North Sydney, NSW 2060, Australia; ^4^Discipline of Pathology, Sydney Medical School, The University of Sydney, NSW 2006, Australia

## Abstract

Well differentiated liposarcoma (WDLS) is the commonest subtype of liposarcoma. Recognised subtypes of WDLSs are lipoma-like, sclerosing, spindle cell and inflammatory. The inflammatory variant of WDLS also known as “lymphocyte-rich liposarcoma” is rare. We present a case of inflammatory WDLS occurring in the retroperitoneum, in a patient with a past history of non-Hodgkin lymphoma. We outline the histological features, discuss the differential diagnoses and highlight the diagnostic pitfalls in interpretation of this lesion on fine needle biopsy.

Well-differentiated liposarcoma (WDLS) is the commonest subtype of liposarcoma, accounting for 40%–45% of all cases [1]. Because WDLS does not metastasize unless dedifferentiation supervenes, it has recently been suggested that the term atypical lipomatous tumour be applied to those tumours occurring in superficial locations amenable to complete excision [1]. The most frequent site for WDLS is in the deep soft tissue of the thigh, followed by the retroperitoneum, paratesticular region, and mediastinum [1]. In the current WHO classification, recognised subtypes of WDLS are lipoma-like, sclerosing, spindle cell, and inflammatory [1]. The inflammatory variant of WDLS, also termed as “lymphocyte-rich liposarcoma,” is rare, accounting for only 2% of all liposarcomas in reported series [2, 3]. In this paper, we present a case of inflammatory WDLS occurring in the retroperitoneum, in a patient with a past history of non-Hodgkin lymphoma, discuss the differential diagnosis, and highlight diagnostic pitfalls.

In 2005, a 67-year-old man of Uruguayan origin, with a background history of ischaemic heart disease, psoriasis, and reflux oesophagitis, underwent resection of a retroperitoneal inflammatory and sclerosing WDLS after investigation of abdominal pain. In 2006, diffuse large B cell lymphoma (DLBCL) was subsequently diagnosed in the femoral head and left ilium and treated with radiotherapy. In 2008 he experienced left-sided abdominal pain and was found to have recurrent nodules in the left renal bed. A computed tomography (CT) abdominal scan showed a mass in the surgical bed of the retroperitoneum containing multiple soft-tissue nodules and fat, suggestive of recurrent retroperitoneal liposarcoma. A fine needle biopsy (FNB) showed a mixed population of lymphoid cells consisting predominantly of medium-sized and large cells with a lesser number of small lymphocytes (Figure 1(a)). The medium-sized and large cells had open chromatin and multiple small nucleoli with a small amount of cytoplasm and showed occasional mitotic figures. Tingible body macrophages were present (Figure 1(b)). No material was present in the cell block preparation. The flow cytometry showed reactive T cells, and two populations of B cells including a normal B cell population with CD19+kappa/lambda ratio of 1.0, and also a larger B cell population showing increased percentage of CD10+/CD19+ cells (17.3%) without any evidence of clonality. In view of the prominence of medium- to large-sized lymphoid cells seen, the cytological appearances were reported as an atypical lymphoid infiltrate (suspicious for recurrent non-Hodgkin lymphoma). However, because the clinical presentation and radiology were both highly suggestive for recurrent liposarcoma, a laparotomy was performed for removal of the retroperitoneal mass. Intraoperatively, two separate but adjacent tumours were identified. The first was adherent to the splenic flexure of the colon and spleen whilst the second was inferior to the first mass. 

Macroscopically, the specimen consisted of a partially encapsulated, circumscribed mass of fatty tissue 130 × 90 × 40 mm. Serial slicing showed that it had a variegated nodular appearance with fibrous septa. Centrally there was light yellow, mostly uniform, fatty tissue, whilst peripherally there were cream-coloured firm nodules measuring from 5 to 12 mm across, some of which coalesced (Figure 2). On microscopy, the tumour had characteristic histopathological features of a well-differentiated, partly sclerosing liposarcoma. The adipocytes were of varying size with occasional typical lipoblasts (Figure 3(a)). Broad fibrous bands were present containing atypical spindle cells (Figure 3(b)) and a mild plasma cell infiltrate. These components merged fairly abruptly with the adjacent peripheral nodules (Figure 3(c)). The latter included a dense lymphoid infiltrate composed of reactive lymphoid follicles admixed with numerous plasma cells (Figure 3(d)). Larger nodules included numerous plasma cells (demonstrated to be polyclonal in nature by positivity with kappa and lambda immunostains) with smaller numbers of admixed B, and T lymphocytes, eosinophils, and scattered larger cells some with a Reed-Sternberg-like appearance (Figures 3(e) and 3(f)). Intermixed fibroblast-like spindle cells were also present. The overall appearances were consistent with recurrent inflammatory (lymphocyte-rich) WDLS. There were no areas of dedifferentiation, and no lymphoma was identified. The patient is currently well, with no recurrence two years after surgical excision of the recurrent tumour.

Most cases of inflammatory liposarcoma have been reported in the retroperitoneum. However, individual case reports of inflammatory liposarcoma have also been described in the pleura [4], scrotum [5], and the spermatic cord [6]. Within the retroperitoneum, the natural history of inflammatory liposarcomas is similar to other WDLS and typically is one of multiple recurrences. This is in part related to the difficulty in entirely resecting the tumour, as well as the tongue-like extension of tumour into the surrounding tissue. 

FNB of the left renal bed in the current case was interpreted as an atypical lymphoid infiltrate (suspicious for recurrent non-Hodgkin lymphoma) rather than recurrent liposarcoma, probably because the FNB represented only sampling of the lymphoid component. In particular, it likely represented sampling of reactive germinal centres, giving rise to a seemingly predominant population of large lymphoid cells. The background history of DLBCL probably also biased the cytological interpretation. The distinction between non-Hodgkin lymphoma and inflammatory liposarcoma is important because the management and prognosis of both entities are so distinctly different that misdiagnosis may lead to an adverse outcome. 

In view of the microscopic features of the cellular foci of inflammatory WDLS, possibilities such as inflammatory myofibroblastic tumour, lymphoma (either Hodgkin or non-Hodgkin), or Castleman's disease may enter the differential diagnosis. Unless the atypical lipomatous component is identified and the adipocytic nature of the tumour recognised, misdiagnosis is likely to occur. If the lipomatous component is obscured by the intense inflammatory infiltrate (which may range from less than 10% to 80% of the total aggregate of the tumour) [3] or the components are poorly sampled at initial macroscopic examination, there is increased risk of misdiagnosis. As well illustrated by our case, FNB specimens providing only a limited sample are likely to be particularly prone to misdiagnosis especially if the reporting pathologist is unfamiliar with the histological features of inflammatory WDLS.

Other differential diagnoses that may be considered microscopically include sclerosing mesenteritis and retroperitoneal fibrosis. In contrast to inflammatory WDLS, the former is characterised by a combination of fibrosis, chronic inflammation, and fat necrosis. The latter, as the name suggests, shows fibrosis encasing retroperitoneal structures, with admixed lymphocytes and plasma cells. Both of these disorders lack the large hyperchromatic cells and lipoblasts of WDLS. It is not surprising that inflammatory WDLS can be confused with inflammatory myofibroblastic tumour, as cellular foci in the former may have a similar appearance. Inflammatory myofibroblastic tumour lacks the atypical adipocytic component of WDLS and shows positive ALK staining in approximately 50% of cases. The so-called inflammatory malignant fibrous histiocytoma (undifferentiated pleomorphic sarcoma) may also be considered in the differential diagnosis of inflammatory WDLS. However, the spindle cells of the latter show less cytological atypia. Thorough sampling of the different regions of the tumour to facilitate identification of an atypical lipomatous component should enable a diagnosis of inflammatory WDLS to be established.

The recognition of inflammatory WDLS is difficult on limited sampling specimens such as FNB samples particularly if the atypical adipocytic component is not sampled or recognised. However, in many instances, awareness of this rare variant of WDLS, knowledge of its pathological features, and careful clinicopathological correlation will enable accurate diagnosis. 

## Figures and Tables

**Figure 1:**
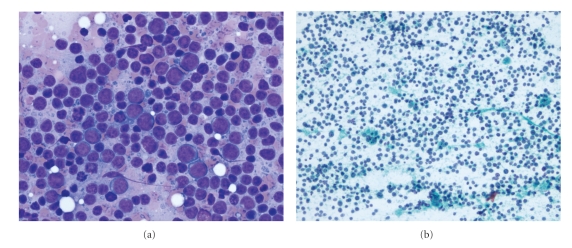
(a) FNB showing a mixed population of lymphoid cells with a predominance of intermediate to large lymphoid cells (DQ). (b) FNB showing a mixed population of lymphoid cells including occasional tangible body macrophages (PAP).

**Figure 2:**
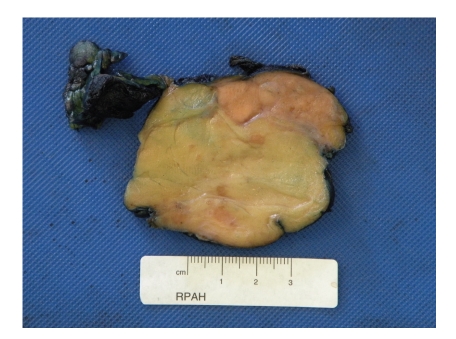
Macroscopic photograph showing a light yellow nodular appearance centrally, with peripheral cream to light brown nodules.

**Figure 3:**
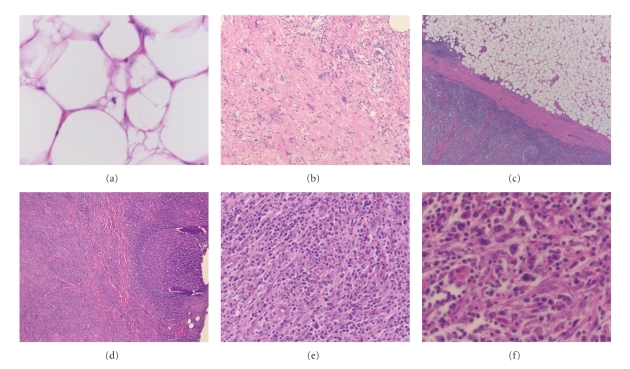
(a) Well-differentiated liposarcoma with occasional lipoblasts (H&E). (b) Broad fibrous bands contained atypical cells and a mild plasma cell infiltrate (H&E). (c) The sharp interface between the well-differentiated liposarcoma component and the lymphoid component (H&E). (d) Peripheral nodules composed of a dense lymphoid infiltrate containing reactive germinal centres and large numbers of chronic inflammatory cells (H&E). (e) The nodules were rich in plasma cells with admixed lymphocytes and eosinophils (H&E). (f) Within this chronic inflammatory infiltrate were scattered larger cells with a Reed-Sternberg-like appearance (H&E).

## References

[1] Dei Tos AP, Pedeutour F, Fletcher CDM, Unni KK, Mertens F (2002). Atypical lipomatous tumour / well differentiated liposarcoma. *Pathology and Genetics of Tumours of Soft tissue and Bone*.

[2] Argani P, Facchetti F, Inghirami G, Rosai J (1997). Lymphocyte-rich well-differentiated liposarcoma: report of nine cases. *American Journal of Surgical Pathology*.

[3] Kraus MD, Guillou L, Fletcher CDM (1997). Well-differentiated inflammatory liposarcoma: an uncommon and easily overlooked variant of a common sarcoma. *American Journal of Surgical Pathology*.

[4] Iqbal M, Posen J, Bhuiya TA, Lackner RP, Steinberg HN, Rossoff LJ (2000). Lymphocyte-rich pleural liposarcoma mimicking pericardial cyst. *Journal of Thoracic and Cardiovascular Surgery*.

[5] Sawazaki H, Nakamura E, Hoshi A (2006). Well-differentiated inflammatory liposarcoma occurring in the scrotum: a case report. *Acta Urologica Japonica*.

[6] Böthig R, Baumunk V, Rosenthal V, Lehmann G (2002). Inflammatory liposarcoma of the spermatic cordInflammatorisches liposarkom des samenstrangs. *Aktuelle Urologie*.

